# Determination of 5-nitro-2-furaldehyde as marker residue for nitrofurazone treatment in farmed shrimps and with addressing the use of a novel internal standard

**DOI:** 10.1038/s41598-019-55809-0

**Published:** 2019-12-17

**Authors:** Qiang Wang, Xu-Feng Wang, Yong-Yuan Jiang, Zhi-Guang Li, Nan Cai, Wan-Qi Guan, Ke Huang, Dong-Hao Zhao

**Affiliations:** 1 South China Sea Fisheries Research Institute, Chinese Academy of Fishery Sciences, Guangzhou, 510300 China; 2 Key Lab. of Aquatic Product Processing, Ministry of Agriculture and Rural Affairs, Guangzhou, 510300 China; 3 Guangdong Provincial Key Laboratory of Fishery Ecology and Environment, Guangzhou, 510300 China

**Keywords:** Analytical chemistry, Medicinal chemistry

## Abstract

We developed a significantly improved ultra-high performance liquid chromatography-tandem mass spectrometry method for determination of 5-nitro-2-furaldehyde (NF) as a surrogate using a novel internal standard for the detection of nitrofurazone. We used 2,4-dinitrophenylhydrazine derivatization and furfural as the internal standard. Derivatization was easily performed in HCl using ultrasonic manipulation for 5 min followed by liquid extraction using ethyl acetate. The samples were concentrated and purified using reverse phase and alumina cartridges in tandem. The derivatives were separated using a linear gradient elution on a C_18_ column with methanol and water as the mobile phase in negative ionization mode and multiple reaction monitoring. Under the optimized conditions, the calibration curves were linear from 0.2 to 20 μg/L with correlation coefficients >0.999. Mean recoveries were 80.8 to 104.4% with the intra- and inter-day relative standard deviations <15% at spiking levels of 0.1 to 10 μg/kg. The limits of detection and quantification were 0.05 and 0.1 μg/kg, respectively. This method is a robust tool for the identification and quantitative determination of NF in shrimp samples.

## Introduction

Nitrofurazone (5-nitro-2-furaldehyde semicarbazone, NFZ) is a broad-spectrum nitrofuran antibiotic frequently used in aquaculture and animal husbandry for treatment of protozoan and bacterial infections^1–3^. Nitrofurans are potential carcinogens and mutagens and China, Japan and the European Union (EU) have banned the use of these drugs in animal husbandry^4,5^. However, nitrofurans are still commonly used in clinical veterinary medicine in some developing countries due to their excellent bactericidal activity and low price^6,7^.

Nitrofurans are rapidly metabolized and localize to tissues within a few hours of administration^8,9^. The most stable metabolite is semicarbazide (SEM) and its detection in tissues is used as evidence for NFZ exposure^10^. The minimum requirement performance limit (MRPL) for SEM in poultry meat and aquaculture products has been set at 1 μg/kg by the EU^11^. The standard detection procedure for SEM is tissue release by acid (HCl) hydrolysis and derivatization with 2-nitrobenzaldehyde. The nitro-phenyl derivatives are detected using liquid chromatography-tandem mass spectrometry (LC-MS/MS)^12,13^. However, SEM derivatives may also occur by reaction with azodicarbonamide and biurea, chemicals commonly used for food preservation^1,14^. In addition, SEM is naturally present in the shells of crayfish, shrimp, prawn and soft-shell crab^15–17^. These data indicate that the use of SEM as the exclusive marker for NFZ might be unreliable^18,19^.

**Figure 1:**
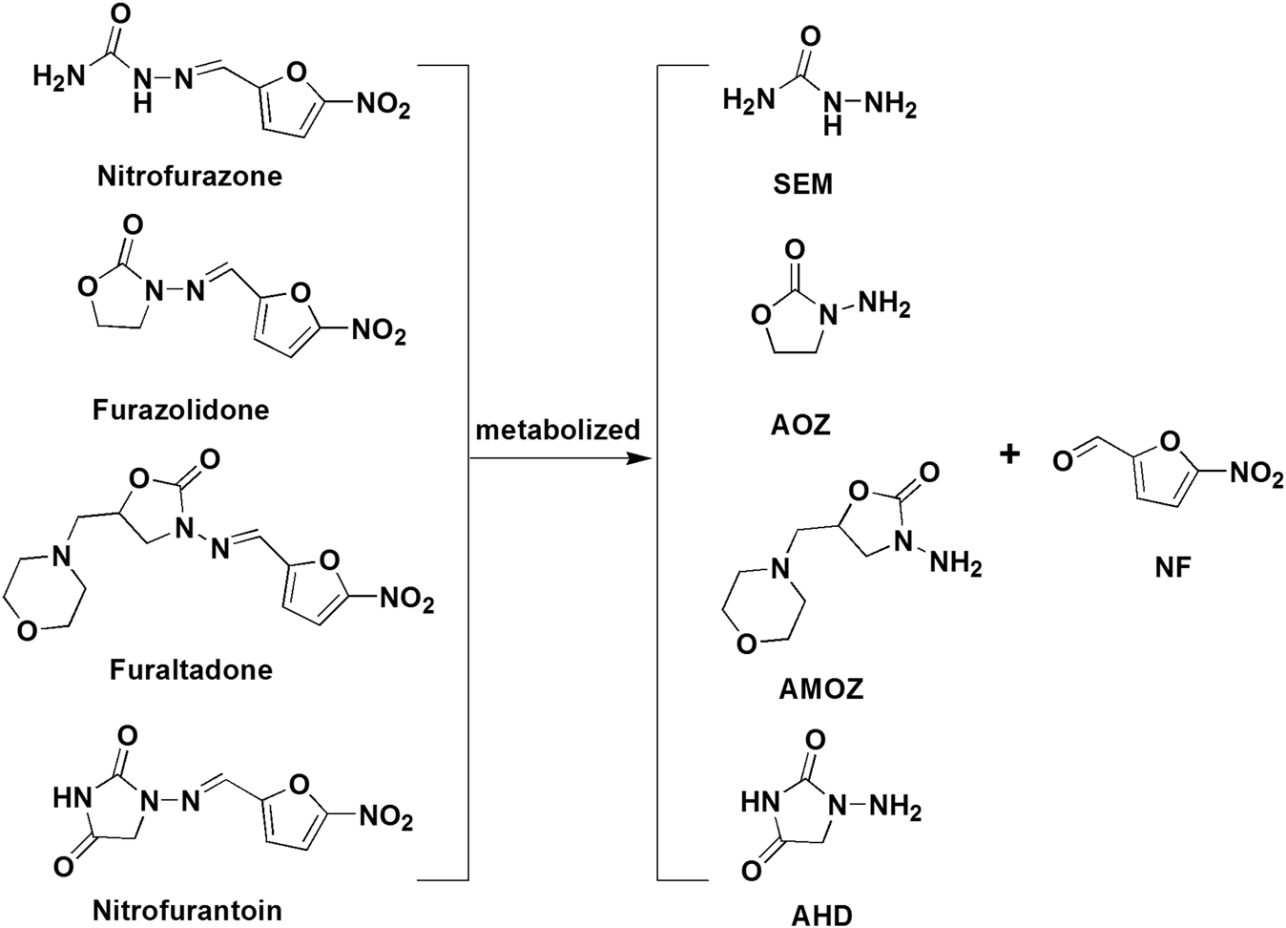
Chemical structures of 4 nitrofurans and its major metabolites.

Another marker for nitrofurazone is 5-nitro-2-furaldehyde (NF), which was first used for NFZ detection in shellfish^20^. However, NF is not a unique maker metabolite to NFZ alone. NF can also be derived from other nitrofuran drugs (such as nitrofurantoin, furaltadone and furazolidone), which have their specific marker metabolite (AHD, AMOZ and AOZ) (Fig. 1). Thus, to avoid the false-positive result induced by SEM alone, further determination of NF should be a selective confirmatory method for NFZ. As an side chain of NFZ, NF can be derivatized with 2,4-dinitrophenylhydrazine (DNPH) for LC-MS/MS analysis. LC-MS/MS coupled with derivatization has advanced analytical techniques in food analysis by increasing sensitivity and selectivity for a wide array of compounds especially when used with internal standards^21^. Internal standards are generally structural and chemical analogs of the target analyte^22,23^. Previous methods for NF determinations have used *p*-dimethylaminobenzaldehyde (PDAB) as an internal standard^24^. PDAB can be derivatized by DNPH but the polarity and reactivity of PDAB differs from NF due to the presence of an additional benzene ring and a dimethylamino substituent. NF possesses a furan ring that is also present in other furfural compounds, such as furfural (FU) and 5-methylfurfural (5-MF).

The aim of this study was to develop a sensitive and reliable UPLC-MS/MS analytical method for the determination of the NF marker after treatment of shrimps by nitrofurazone. FU-D4, FU and 5-MF were used as novel candidate internal standards. We optimized the extraction solvent, the derivatization reaction time, temperature and HCl concentration to obtain maximal sensitivity and accuracy. We also added a solid-phase extraction (SPE) step to minimize matrix interference. The proposed method was validated by the analysis of NF in both freshwater and marine shrimp samples.

## Results

### Optimization of the mass spectrometry conditions

**Figure 2:**
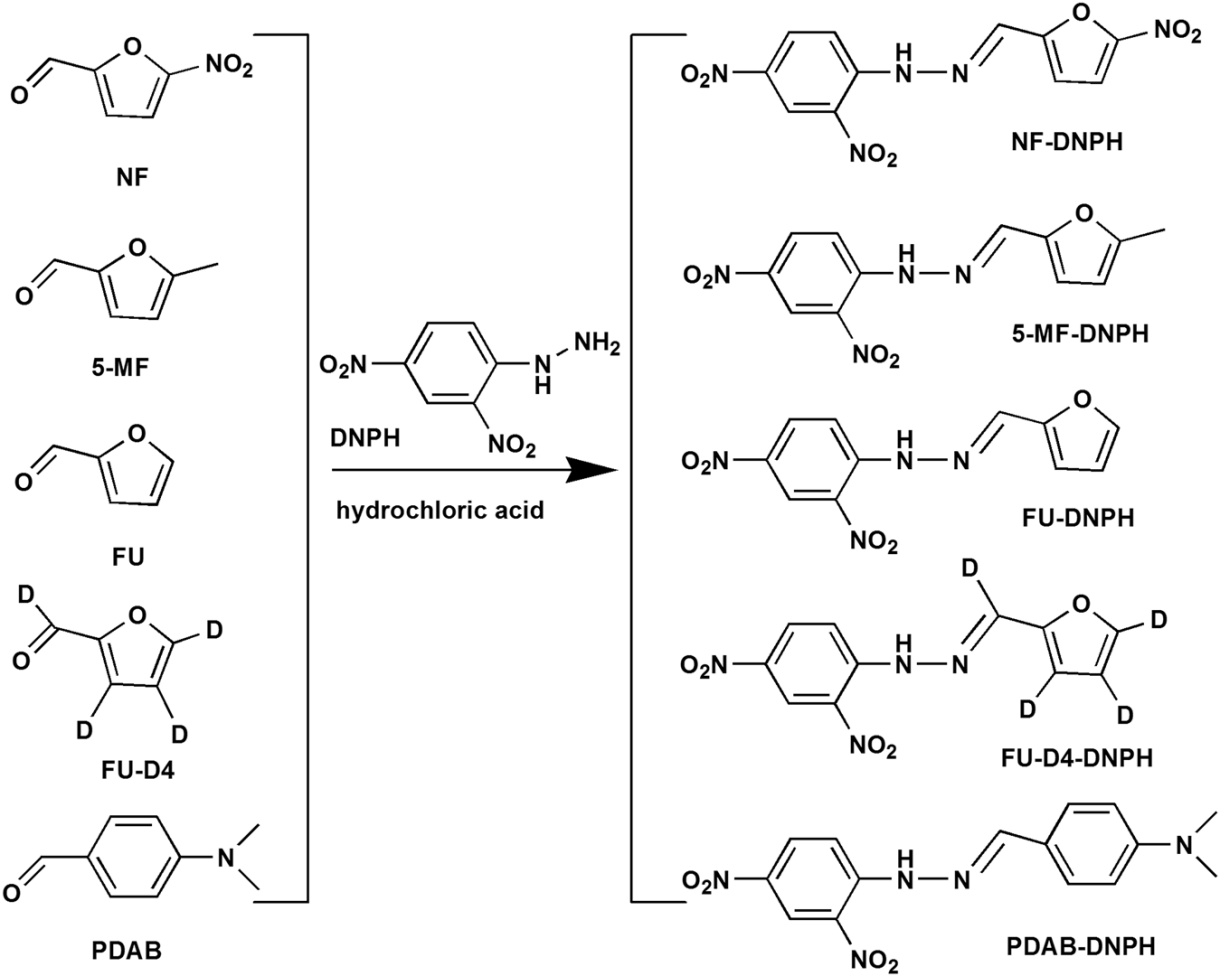
Synthesis routes of DNPH derivatives.

NF analytes and candidate internal standards FU, FU-D4, 5-MF and PDAB were derivatized using acidic DNPH (Fig. 2). Aldehyde carbonyls reacted with DNPH amino groups forming the corresponding DNPH derivatives^25,26^. The yellow DNPH derivatives that were formed were concentrated and kept in MeOH prior to MS.

**Figure 3:**
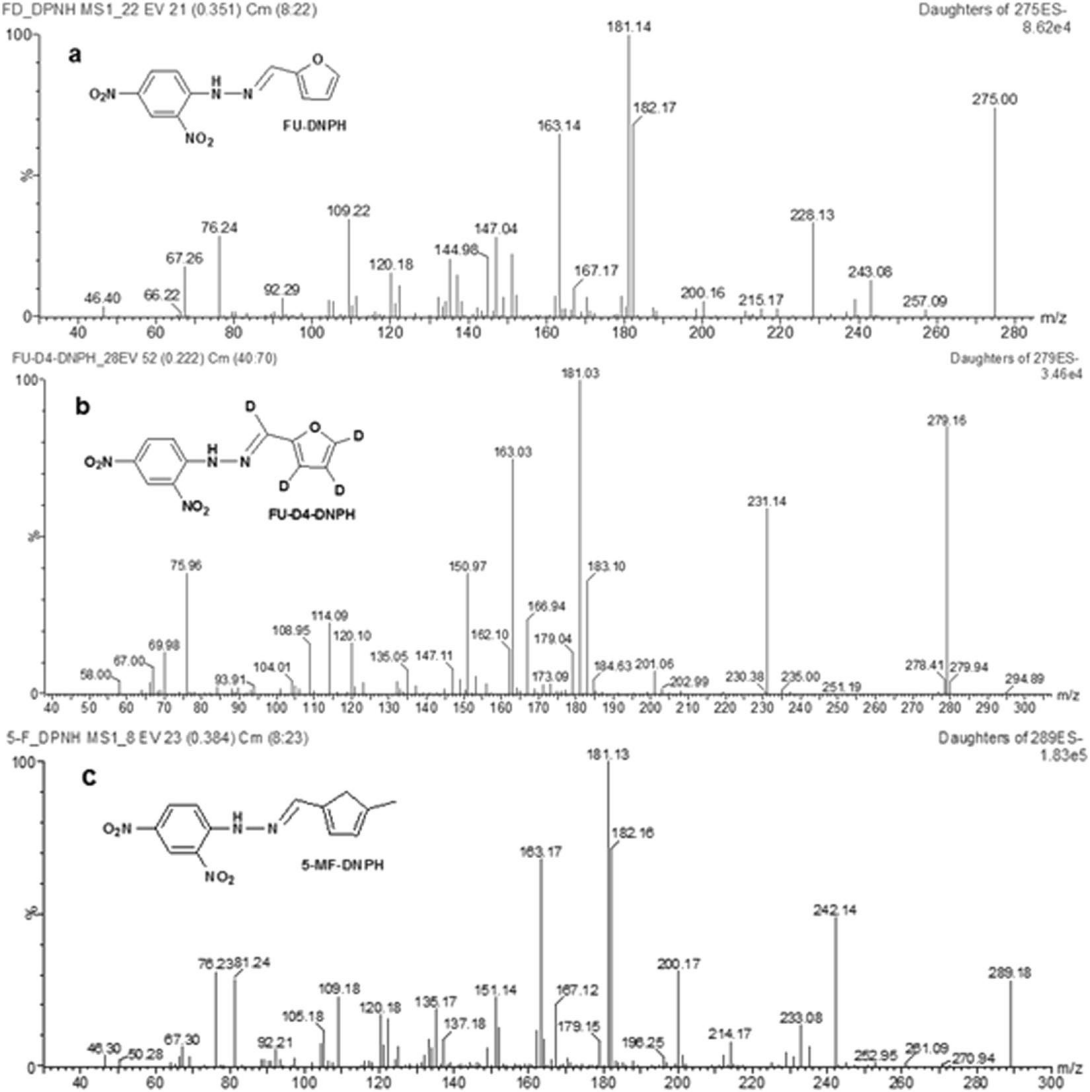
MS/MS spectra of FU-DNPH (**a**), FU-D4-DNPH (**b**) and 5-MF-DNPH (**c**) under daughter ion scan mode (see text for details).

MS parameters were optimized by infusing each DNPH derivative solutions at 0.01 mL/min. Precursor ions were identified in the full-scan mode from *m/z* 50 to *m/z* 500 in ESI^−^ mode and deprotonated molecular ions [M–H]^−^ were selected for all analytes. The DNPH derivatives of NF, FU, FU-D4, 5-MF and PDAB produced stable precursor ions at *m/z* 320.1, 275.0, 279.1, 289.2 and 328.2, respectively. The NF-DNPH and PDAB-DNPH ions were consistent with the results of a previous study^24^. FU-DNPH, FU-D4-DNPH and 5-MF-DNPH were further identified using daughter scans at different collision energies (5 to 40 eV). FU-DNPH could fragment into two characteristic ions of *m/z* 228.1 (M-NO_2_) and *m/z* 181.1 (a further loss of −NO_2_). Cleavage pathways of FU-D4-DNPH and FU-DNPH were analogous by negative electrospray ionization mass spectrometry. Correspondingly, 5-MF-DNPH produced product ions of *m/z* 242.1 (M-NO_2_) and *m/z* 181.1 (a further loss of −NO_2_ and −CH_3_) (Fig. 3). Quantification was performed using an MRM experimental setup (Table 1).

**Table 1:** Transitions and optimal conditions used for MS/MS (^a^Transition ions for quantification).

Compound	Retention time (min)	Precursor ion (*m/z*)	Transition ions (*m/z*)	Cone voltage (V)	Collision energy (V)	Ion ratios
NF-DNPH	3.65	320.1	273.2^a^, 161.2	2	14, 18	0.48
5-MF-DNPH	3.89	289.2	242.2, 181.1^a^	10	16, 20	0.82
FU-DNPH	3.71	275.0	228.1, 181.1^a^	44	12, 30	0.96
FU-D4-DNPH	3.71	279.1	231.1, 181.1^a^	50	22, 34	0.95
PDAB-DNPH	4.27	328.2	281.3^a^, 163.3	40	16, 18	0.36
DNPH	2.40	197.2	151.2^a^, 121.2	24	8, 22	0.52

### Optimization of LC chromatographic conditions

**Figure 4:**
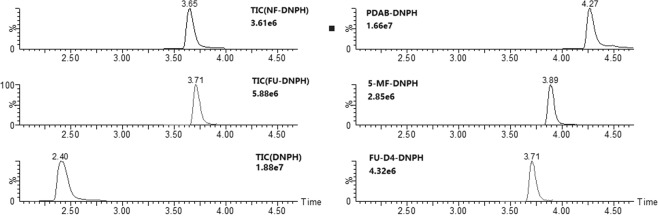
MRM chromatograms of DNPH and five DNPH derivatives at 10 μg/L.

We optimized LC conditions by comparing ACN and MeOH as the organic component. The used of MeOH increased the sensitivity with better peak shapes and greater resolution for most of the analytes. We also tested the addition of ammonium acetate (5–10 mM) to the water phase as a counterion but could not detect any obvious differences. Thus, we started the optimization procedure with an elution gradient of water and MeOH. When the MeOH component exceeded 90%, the DNPH derivatives were eluted completely. Using the optimized chromatographic conditions, retention times of 3.65 to 3.89 min were obtained for NF-DNPH, 5-MF-DNPH, FU-DNPH and FU-D4-DNPH. PDAB-DNPH eluted at 4.27 min most likely due to its additional benzene ring. In contrast, DNPH was eluted at 2.4 min at 50% MeOH (Fig. 4).

### Effect of extraction solvent

**Figure 5:**
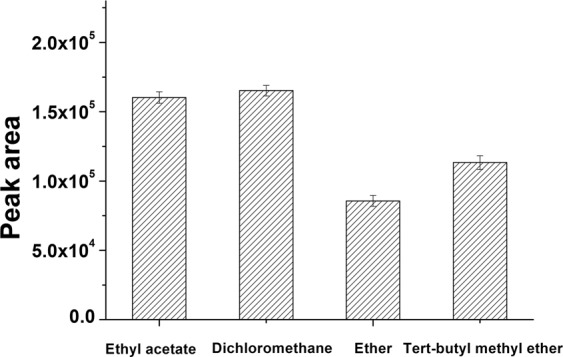
Comparative peak areas of NF-DNPH extracted from spiked shrimp matrix using the indicated solvents. Each bar represents the average peak areas and standard deviations of three replicates.

Derivatized solutions were adjusted to ~pH 7 and we compared extraction efficiencies using ethyl acetate, dichloromethane, diethyl ether and tertbutyl methyl ether. The experiment was performed in the presence of the shrimp matrix. We found no obvious evidence for emulsification with any of the solvents after centrifugation. The extraction efficiencies of diethyl and tert-butyl methyl ether for NF-DNPH were relatively low. Ethyl acetate and dichloromethane were more appropriate solvents for NF-DNPH extraction (Fig. 5). In view of the high toxicity of dichloromethane, ethyl acetate was chosen as the extraction solvent for the remainder of this study.

### Effect of derivatization conditions

**Figure 6:**
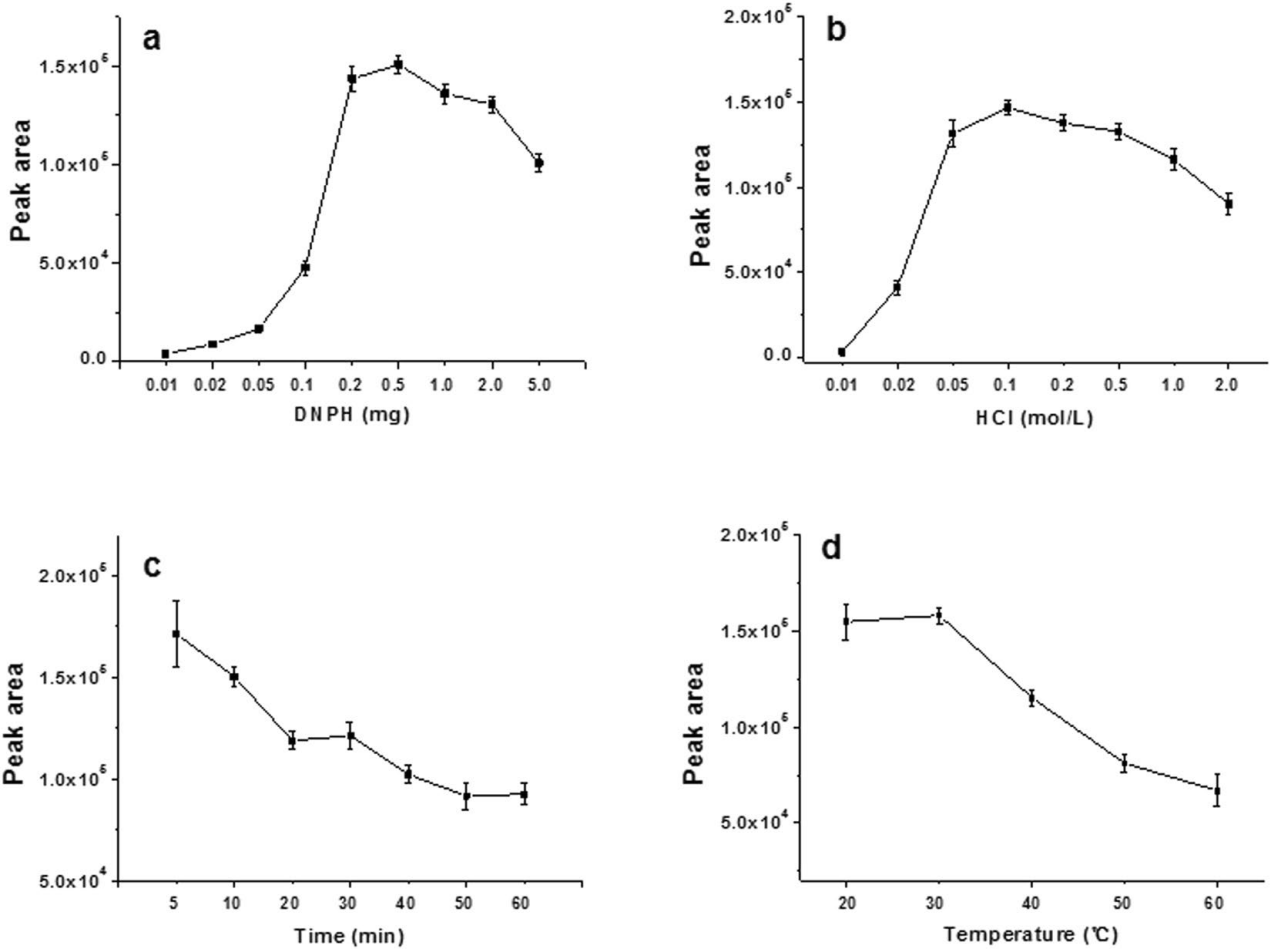
Influence of different reaction components on NF-DNPH quantification. (**a**) DNPH (**b**) HCl (**c**) reaction time (**d**) temperature. Each point represents the average peak area and standard deviation of three replicates.

In the absence of matrix interference, aldehydes react easily with DNPH in acidic solutions to produce derivatives at equal molar ratios. The introduction of a tissue matrix introduces another level of complexity into the extraction procedure. The primary cause of incomplete derivatization is insufficient DNPH so we investigated whether DNPH levels were sensitive to matrix effects. DNPH was added at 0.01 to 4 mg per reaction using 10 ng NF and 2 g homogenized shrimp. When the reaction system contained 0.01 mg DNPH, the UPLC-MS/MS signal was almost totally absent. NF-DNPH increased as the amount of DNPH was increased and the largest peak area of NF-DNPH was observed at 0.5 mg DNPH and further addition did not increase the yield (Fig. 6a). Thus, we used 0.5 mg DNPH for derivatization in the presence of 2 g shrimp matrix.

The concentration of HCl could also affect reaction efficiency so we varied HCl from 0.01 to 2M. We found that 0.1M HCl was optimal for the derivatization and extraction processes (Fig. 6b).

The effects of time and temperature on derivatization efficiency were also examined. NF reacts rapidly with DNPH and the largest peak area of NF-DNPH was observed within five minutes and declined after this (Fig. 6c). The greatest sensitivity was obtained when the derivatization was performed at 30 °C after checking the range of 20–60 °C (Fig. 6d). A prolonged reaction time and higher temperatures most likely generated interfering impurities.

### Selection of internal standard

Four candidate internal standards (FU, FU-D4, 5-MF and PDAB) were examined in the optimized reaction conditions at 10 ng each in 2 g shrimp samples containing 10 ng NF. The derivatives FU-DNPH, FU-D4-DNPH, 5-MF-DNPH and PDAB-DNPH were individually used as internal standards for quantitative analysis. The recoveries of NF-DNPH in the presence of each of these compounds were 94.6%, 92.5%, 83.8% and 141%, respectively. PDAB was not a suitable internal standard for NF due to its poor accuracy most likely due to similarities in chemical structure. FU and FU-D4 can be recommended as internal standard for NF calibration. However, the hygroscopic isotope internal standard of FU-D4 was too expensive. Thus, FU was selected as internal standard in subsequent experiments.

### Optimization of SPE procedures

**Figure 7:**
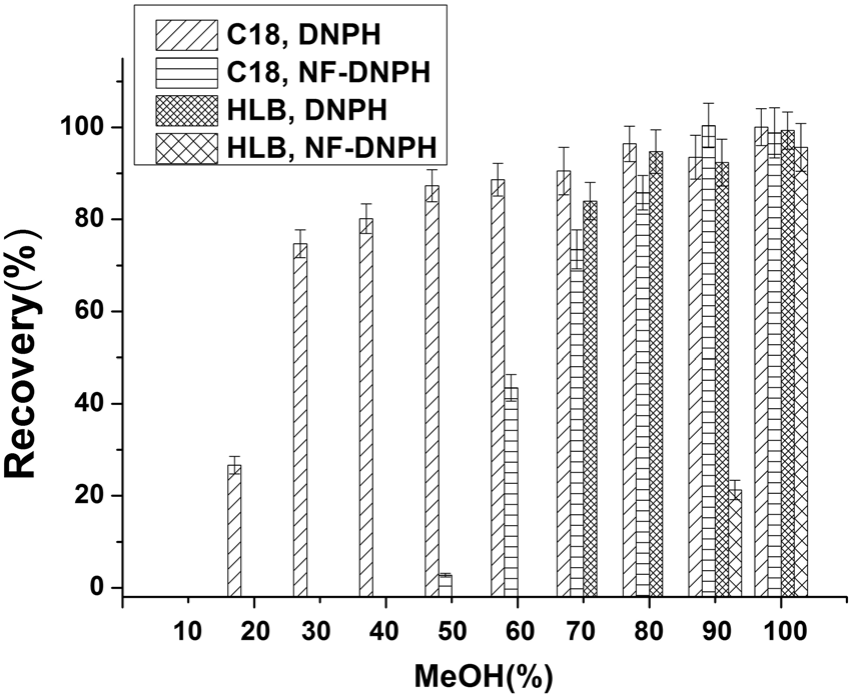
Recoveries from SPE cartridges after altering MeOH (%) in water as the elution solvent. Each bar represents the average peak areas and standard deviations of three replicates.

Ethyl acetate is a highly selective solvent for nonpolar compounds and these include fat and other matrix impurities. Thus, an additional extraction step was necessary and we introduced an SPE step to minimize matrix interference. Previous studies had relied on the universal reversed-phase HLB cartridge for removal of phospholipids, fats, and colored substances from aquatic products^24,27^. This method involved the loading of 1 mL HCl onto the cartridge and the effluent was used for UPLC-MS/MS^24^. However, HCl affects ionization efficiency in the ESI source using the negative ion mode. We therefore tested a C_18_ cartridge (200 mg, 3 mL), an HLB cartridge (60 mg, 3 mL) and a neutral alumina cartridge (1 g, 3 mL) to develop and optimize the SPE method. All cartridges were conditioned successively with 3 mL MeOH and 3 mL water, and were washed with 3 mL water and eluted with 3 mL MeOH. The HLB and C_18_ cartridges produced satisfactory recoveries (>95%) for NF-DNPH whereas the neutral alumina cartridges did not adsorb NF-DNPH. Thus, the neutral alumina cartridges could be used in tandem with a reverse phase cartridge for removal of fats and other granular impurities as a further clean-up procedure. The eluent color following SPE treatment was almost identical to the yellow extraction containing excessive DNPH and meant that the derivative reagent was not removed. We therefore designed an elution experiment for optimizing the rinse and desorption conditions. The reverse phase cartridges were tested using different MeOH-water solutions (10–100%). As the MeOH content was increased, NF-DNPH was not eluted. Almost 80% DNPH was rinsed from the C_18_ cartridge using 3 mL 40% MeOH while retaining NF-DNPH (Fig. 7). In contrast, 3 mL of 80% MeOH washed out most DNPH but no NF-DNPH using the HLB cartridge. The HLB cartridge better served our purification purpose and was used in tandem with a neutral alumina cartridge for the remainder of the purification procedures.

### Matrix effects

Matrix effects were evaluated by comparing the MRM chromatograms of test compounds in a shrimp homogenate with and without SPE purification. The coefficient between the slopes of matrix-matched and solvent-based standard curves was 0.918 and indicated a tolerable weak matrix suppression effect in this study. The peak areas of NF-DNPH and FU-DNPH increased over 1.5 fold following SPE clean-up due to the diminished matrix suppression effect.

### Linearity, LOD, LOQ, accuracy and precision

The NF-DNPH concentrations in shrimp samples were estimated using FU-DNPH as the internal standard. A satisfactory linearity was achieved in the range of 0.2 to 20 μg/L with a 1/x weighting factor and the resulting correlation coefficients (R^2^) were >0.999. Both freshwater (*Macrobrachium rosenbergii*) and marine (*Litopenaeus vanname*i) shrimp samples were used to verify the accuracy and precision of the method. Blank shrimp samples at four spiking levels of 0.1, 0.5, 2 and 10 μg/kg were prepared for the SPE-UPLC-MS/MS analysis. Mean recoveries ranged from 80.8% to 104.4% with the intra- and inter-RSDs <15% (Table 2). The LOD and LOQ of NF in shrimp samples were 0.05 and 0.1 μg/kg, respectively, which were slightly lower than a previous study^20^. Generally speaking, the SPE-UPLC-MS/MS method we developed satisfied the quantitative analysis of NF at trace levels in shrimp samples.

**Table 2:** Accuracy and precision of NF from spiked shrimp samples (n = 5).

Sample	Spiked (μg/kg)	Recovery (%)	Intra-day RSD (%)	Inter-day RSD (%)
*Macrobrachium rosenbergii*	0.1	104.4	10.3	12.5
	0.5	86.1	9.1	5.8
	2.0	90.2	5.1	7.4
	10.0	92.3	7.7	10.2
*Litopenaeus vannamei*	0.1	80.8	6.2	8.1
	0.5	95.4	9.5	11.3
	2.0	91.4	4.8	9.7
	10.0	89.7	5.3	6.5

### Method application

In the previous report, metabolites analysis results of NF and SEM in real representative samples were compared, and the marker metabolite of NF could be used for identification of the real NFZ-abuse sample^20^. The applicability of the developed method has been evaluated by analyzing NF in 30 shrimp samples collected from a local aquaculture farm. NF was found in four samples at concentrations of 1.04–3.87 μg/kg. The SEM residues in these samples was also determined in accordance with the Announcement No. 783-1-2006 of the Ministry of Agriculture of the People’s Republic of China^28^. The NF residue was less than that of SEM at concentrations of 3.57–15.61 μg/kg in shrimp. In addition, shells collected from shrimp that were not exposed to NFZ were also detected. The results of SEM were positive, but NF could not be detected.

## Conclusions

A significantly improved UPLC-MS/MS method was developed for the determination of NF in shrimp using DNPH derivatization. FU was selected as the internal standard and we obtained better sensitivity and accuracy than previously used internal standards. The sample pre-treatment methods including derivatization, SPE and sample preparation were also optimized. The practical method produced a low LOQ (0.1 μg/kg) with excellent recoveries and satisfactory precision. This method is ideally suited for monitoring the illegal use of nitrofurazone in aquatic products.

## Materials and Methods

### Chemicals and reagents

NF (purity >99%) was purchased from Alfa-Aesar (Ward Hill, MA, USA). DNPH, FU and 5-MF, all >98% purity, were obtained from Macklin Biochemical (Shanghai, China). PDAB (purity >99%) was purchased from Acros Organics (Geer, Belgium). Furfural-D4 (FU-D4) was purchased from Toronto Research Chemicals Inc. (Toronto, Canada). Neutral alumina cartridges (1 g, 3 mL) were obtained from ANPEL Laboratory Technologies (Shanghai, China). Oasis HLB cartridges (60 mg, 3 mL) were purchased from Waters (Milford, MA, USA). Bond Elut-C_18_ cartridges (200 mg, 3 mL) were supplied by Agilent Technologies (Folsom, CA, USA). HPLC grade methanol (MeOH) and acetonitrile (ACN) were obtained from Fisher Scientific (Pittsburgh, PA, USA). Ultrapure water for all aqueous solutions used for this study was prepared using a Milli-Q reagent water system (Millipore, Bedford, MA, USA).

### Preparation of standard solutions

Standard stock solutions of NF, 5-MF, FU, FU-D4 and PDAB were prepared in MeOH at 1 g/L. Derivatizing solutions (5 g/L) was prepared by dissolving 0.25 g of DNPH in 1 mL 12 M HCl and diluting with MeOH in a 50 mL volumetric flask. All of the stock solutions were kept in brown glass vials and stored at −20 °C. Working standard solutions of 100 and 10 μg/L were prepared by dilution of the stock standard solutions with 50% MeOH.

### Instrumentation

The liquid chromatography system was a Waters Acquity UPLC I-class system equipped with a sample manager, a quaternary solvent pump and a column oven. Mass spectrometry measurements were conducted using a Xevo TQ-S triple-quadrupole mass spectrometer with an electrospray ionization (ESI) source (Waters). Data was processed using MassLynx 4.1 software supplied with the instrument.

### Derivatization with DNPH

Standard stock solutions (1 g/L, 0.5 mL) of NF, 5-MF, FU, FU-D4 and PDAB were dissolved in 10 mL 0.1 M HCl followed by the addition of DNPH (5 g/L, 0.1 mL) and then kept in an ultrasonic water bath for 30 min and then extracted with 10 mL ethyl acetate. The upper yellow organic phase was evaporated to dryness under nitrogen at room temperature and the residue was dissolved in 10 mL MeOH. NF-DNPH, 5-MF-DNPH, FU-DNPH, FU-D4-DNPH and PDAB-DNPH were analyzed by mass spectrometry.

### Sample preparation

Shrimp shells were removed and the internal portions were homogenized and 2 g samples were combined with 10 mL 0.1M HCl in 50 mL polypropylene centrifuge tubes. Aliquots (100 μL each) of FU (100 μg/L) and DNPH (5 g/L) were added successively. The tubes were vortexed for 10 s and immersed in an ultrasonic water bath at 30 °C for 5 min. After cooling to room temperature, the pH of the mixture was adjusted to 7.4 with 0.1 M K_2_HPO_4_ (5.0 mL) and 1 M NaOH (0.2 mL). Ethyl acetate (10 mL) was added and the sample was vortexed for 1 min and centrifuged (4000 × g) at 4 °C for 10 min. The extraction procedure was repeated using the pellets of the centrifugation step. The upper layers containing ethyl acetate were evaporated to dryness in a 40 °C water bath under a stream of nitrogen. The dried extract was prepared for solid phase extraction (SPE) by reconstituting in 0.5 mL MeOH and ultrasonicating for 1 min and followed by the addition of 0.5 mL water.

### SPE

The clean-up procedure was performed using a neutral alumina cartridge linked directly to an HLB cartridge. The cartridges were preconditioned with 3 mL MeOH and 3 mL distilled water and the samples were directly loaded. The cartridges were washed with 3 mL water and 3 mL 80% MeOH successively and eluted with 3 mL MeOH. The eluates were evaporated to near dryness at 40 °C under a stream of nitrogen and residue was dissolved in 1 mL 50% MeOH for UPLC-MS/MS analysis.

### UPLC–MS/MS analysis

The analytes were separated on a Kinetex EVO C_18_ 100 Å column (50 × 2.1 mm, 1.7 μm, Phenomenex, Torrance, CA, USA) at 35 °C. The mobile phase consisted of (A) water and (B) MeOH. The gradient elution program was as follows: 0–3.5 min, 10%–90% B; 3.5–4.5 min, 90% B; 4.5–4.6 min, 90%–10% B; 4.6–6.0 min, 10% B. The injection volume was 10 μL and the flow rate was 0.3 mL/min.

The ionization source conditions were as follows: ESI source, negative mode; capillary voltage, 3.0 kV; source temperature, 150 °C; desolvation temperature, 600 °C; cone gas (N_2_) flow rate, 150 L/h; desolvation gas (N_2_) flow rate, 800 L/h. The collision gas flow was set at 0.15 mL/min. The corresponding collision energy (CE) and cone voltage (CV) values were given in Table 1. Quantitation was performed in multiple reaction monitoring (MRM) mode and the conditions were optimized for each analyte during infusion with their corresponding CE and CV values.

### Method validation

Validation was operated partly, according to the Commission Decision 2002/657/EC^29^ (substance identification and linearity, accuracy and precision) and partly not, especially for the analytical limits (LOD and LOQ instead of CCalpha and CCbeta). A series of standard solutions of NF (0.2–20 μg/L) were employed as working standards for calibration using the internal standard method. Accuracy and precision were validated by the recovery studies. Blank shrimp samples (2 g) were spiked with different volumes of NF standard solution, leading to fortification levels in the range of 0.1–10 μg/kg. The intra- and inter-day precision was evaluated using the relative standard deviation (RSD) of repeated measurements in quintuplicate in a single day and five consecutive days, respectively. For substance identification, two MRM transitions were selected for each analyte giving 4 identification points.

The matrix effect in the present work was calculated by comparing the slope of the calibration curve of the blank extract with that of the pure solvent^30^. The LOD and LOQ values were evaluated by diluting the stock standard solutions with the blank shrimp matrices to the lowest concentration with signal to noise ratio of 3:1 and 10:1, respectively.

## Data Availability

The datasets generated during and analyzed during the current study are available from the corresponding author on reasonable request.
